# TRAP Sequence Clinical Case: Is a Single Umbilical Artery a Protective Ultrasound Marker for Fetal Heart Failure Development

**DOI:** 10.1007/s00246-025-03848-y

**Published:** 2025-04-06

**Authors:** Sakine Rahimli Ocakoglu, Bilge Kapudere, Zeliha Atak, Ozlem Ozgun Uyaniklar

**Affiliations:** 1 Clinic of Obstetrics and Gynecology, University of Health Sciences, Bursa City Hospital, 16110 Nilufer, Bursa Turkey; 2 Clinic of Maternal-Fetal Medicine, University of Health Sciences, Bursa City Hospital, 16110 Nilufer, Bursa Turkey

**Keywords:** Reversed arterial perfusion, Single umbilical artery, Fetal heart failure

## Abstract

Twin Reversed Arterial Perfusion (TRAP) sequence is a rare complication in monochorionic pregnancies, often leading to fetal heart failure (FHF) in the pump twin. We present a 17-year-old primigravida with a monochorionic monoamniotic twin pregnancy presented with an acardiac twin, single umbilical artery (SUA), and multicystic placenta appearance. Throughout the pregnancy, the pump twin exhibited normal growth without signs of FHF. The infant was delivered at 34 weeks via cesarean section, healthy and without cardiac anomalies. The nonexistence of an impacted intrauterine fetal development and FHF of the pump twin in our case may be explained by the presence of one umbilical artery, resulting in less blood flow to the parasitic twin, which can be accepted as a protective factor for the pump twin circulation. Our hypothesis-generating finding that SUA may play a protective role in the development of FHF in pump-twin is promising; however, it remains speculative, and further research is needed to confirm the protective role of SUA for pump-twin outcomes in TRAP pregnancies.

## Introduction

Twin reversed arterial perfusion (TRAP) sequence is a rare and severe complication in monochorionic pregnancies. The approximate incidence of TRAP sequence in monochorionic twin pregnancies is 2.6% [1]. This is a sequence in which a twin with multiple and severe abnormalities (recipient-parasitic twin) known to have an absent or rudimentary heart, also known as an "acardiac twin," is perfused by its co-twin (pump twin). The cardiovascular system of the pump twin (provider twin) provides circulatory support to the acardiac twin from the first trimester onwards. Perfusion, supplied by the provider twin, appears to result from abnormal arterio-arterial anastomosis to the lower half of the recipient twin [2].

Doppler ultrasound confirms the diagnosis, revealing arterial blood flow towards the acardiac twin in its umbilical arteries. This selective perfusion causes various structural abnormalities in the acardiac twin. The upper body and cranium are poorly developed or absent. Umbilical-venous return to the pump twin is conditioned by venovenous anastomosis. Oxygenated blood from the placenta and deoxygenated blood from the acardiac twin lead to volume preload. Therefore, the provider twin may develop high-output cardiac failure (fetal heart failure (FHF) associated with polyhydramnios, cardiomegaly, tricuspid regurgitation, pleural effusions, ascites, and hydrops fetalis [3].

FHF is when the fetal heart fails to provide sufficient blood flow for tissue perfusion in various organs, especially the brain, heart, liver, and kidneys. FHF is associated with inadequate cardiac output, which can lead to premature birth or even intrauterine fetal loss [4]. On the other hand, the deoxygenated blood flow from the acardiac twin may lead to chronic hypoxia and growth restriction in the pump twin. In TRAP pregnancies, the development of FHF depends on the size of the acardiac twin. FHF is not a disease but rather a late consequence of several disorders and can lead not only to fetal morbidity but also cause unfavorable intrauterine programming of an individual future health.

## Case Report

A 17-year-old primigravid woman was referred to our tertiary referral hospital due to an indication of pregnancy under 18 years of age (adolescent pregnancy) for a comprehensive examination and radiological evaluation. The patient's gestational week at first admission was 13 weeks 5 days, and no pathology was found in the patient's "anamnesis vitae" and "anamnesis morbi". Ultrasonography examination (US) revealed a monochorionic monoamniotic (MCMA) twin pregnancy with a viable fetus at 13 weeks 2 days and a fetus with no fetal cardiac activity (FCA) at 10 weeks 1 day. The acardiac fetus has acrania and multiple anomalies; due to US assessment, the patient was referred to the Perinatology and Pediatric Cardiology Department of our hospital for further examination.

**Fig. 1:**
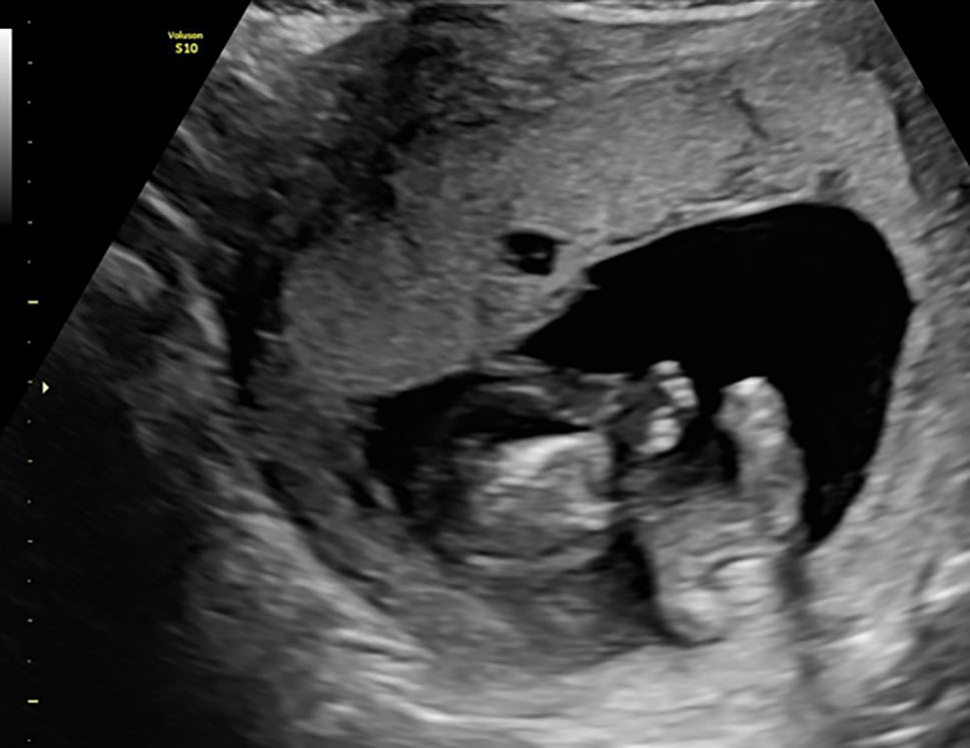
Perinatology team assessment: viable fetus (left): the nasal bone, the stomach, and bladder were observed; The lower and upper extremities were observed as active. No tricuspid valve regurgitation and abnormal waveforms in fetal ductus venous flow assessment were monitored; a single umbilical artery was detected in the fetal umbilical cord. Co-twin (right) shows that the fetus has acrania and acardia, and the upper extremities were not visualized. The spine and lower extremities are observed, but intraabdominal organs could not be detected. Circulation was observed in the acardiac twin, and a Uterine arterial Doppler ultrasound assessment found both the left and right uterine Doppler early diastolic notch

Perinatology clinic assessment shows that according to the last menstrual period (LMP), the patient was 13 weeks and 5 days pregnant. US of perinatology team relevant, viable fetus with CRL measurement in 13 weeks and 4 days, and co-twin CRL measurement in 10 weeks and 4 days, with no fetal cardiac activity, with multiple abnormalities (Fig. 1), and abnormal placental image—multiple cystic placenta appearance. Patient with a preliminary diagnosis of *Twın Pregnancy, MCMA, Trap Sequence, Single Umbilical Artery (SUA), and Placental Mesenchymal Dysplasia (PMD)* was taken under close perinatology clinic observation for further examination. The family was informed about chromosomal diseases, and karyotype analysis was offered. Genetic counseling was recommended. The invasive test, Chorionic villus sampling (CVS), and placenta tissue biopsy were carried out. No abnormalities were found in the CVS material analysis, and the placental biopsy did not detect any pathological findings. The maternal and paternal karyotype results, quantitative fluorescence polymerase chain reaction (QF-PCR), and comparative genomic array hybridization tests were not pathological.

**Fig. 2:**
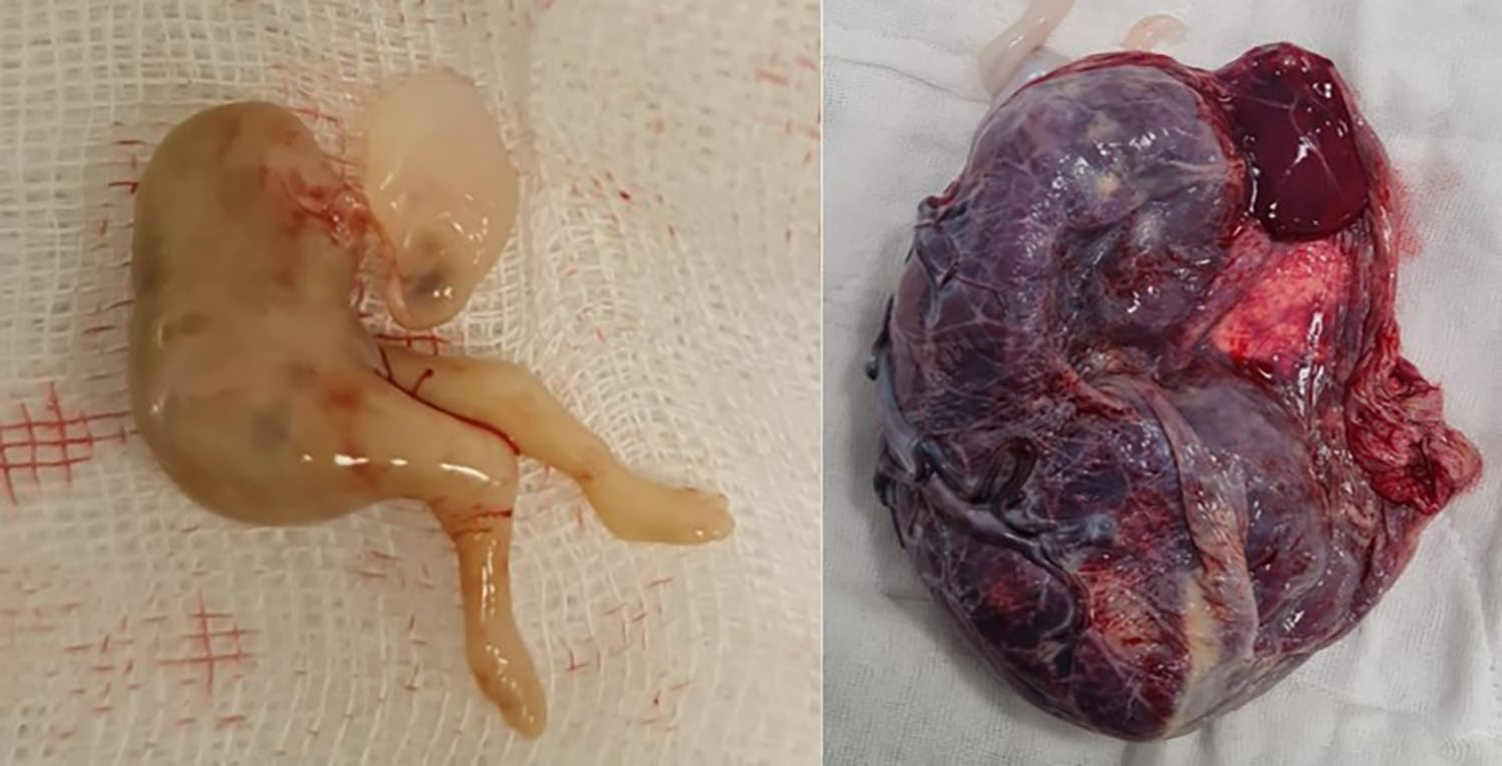
*Left*: histopathological report of fetal specimen: 4.5 × 2 × 1 cm in size; there are findings of acrania, maceration, and autolysis- status *"Acardius acephalus"*. *Right*: histopathological report of placenta specimen: no placentomegaly and 14 × 13 × 10 cm of placental tissue was detected in size. The umbilical cord contained 2 vessels and was 13 cm in length and 1.5 cm in diameter. Decidual tissues, chorionic villus containing subcortical hematoma, and intraparenchymal hemorrhage were detected

The perinatology and pediatric cardiology teams carried out regular pregnancy follow-ups of the patient; during the antenatal period, normal fetal growth and anatomy were observed at provider twin. The pediatric cardiology team did not detect FHF during pregnancy in the provider twin. The patient did not require any emergency obstetric care or hospitalization. No circulation was observed in the acardiac twin at 21 weeks of gestation. At the 34th week of gestation, the patient was delivered by cesarean section following two doses of corticosteroid treatment for fetal lung maturation. A female infant weighing 2035 g was delivered by cesarean section with an Apgar 9–10 score; the pediatric unit found no obvious dysmorphic features and FHF at the first examination of the newborn. In addition, postnatal examination of the child at 3 and 6 months did not reveal any cardiac pathologies. Non-viable fetus and placenta (Fig. 2) were referred to histopathological evaluation. Microscopic evaluation of the placenta did not reveal pathological signs; however, 2 vessels were found in the umbilical cord instead of 3.

## Discussion

The case report of Nicolì P. with the intriguing and short sentence "Intuitively, the earlier the diagnosis, the better the outcome," emphasizes the real picture and seriousness of the TRAP. As it is known, intrauterine cardiovascular circulation differs from postnatal circulation [5]. Having sufficient knowledge in this topic ensures correct approaches to fetal cardiovascular diseases and contributes to a precise determination of postnatal prognosis. Predicting the outcome of pregnancy complicated by TRAP is challenging due to the rarity of this disease and the heterogeneity of its clinical presentation. Sullivan et al. found a less dramatic rate of fetal/neonatal mortality in TRAP pregnancies, with only one case of ten TRAP-diagnosed patients (10%) resulting in the death of the pump fetus, compared with rates of 50% to 75% previously reported [6]. What are the real predictors of good fetal/neonatal outcomes in TRAP pregnancies?

To the best of our knowledge, this is the first reported case combining these three findings: TRAP, SUA, and multiple cystic placenta appearances without pathological abnormalities. The placenta is a pregnancy-related organ developed at implantation of the blastocyst and loses function immediately after delivery of the baby. Proper implantation, vascularization, and placenta development are essential in embryogenesis and fetal growth. In our patient, one of the main findings first noticed upon evaluation by the initial sonographer and perinatology team was the placenta's multicystic appearance and SUA's. The multiple cystic placenta images we found in the US may be related to molar degeneration or PMD [7]. In our case, we sonographically identified aneurysmal dilatation of the vessels on the fetal surface of the placenta, but the biopsy sample does not confirm this PMD finding. In this report, the widespread cystic appearance of the placenta during US screening can be explained by abnormal vascular anastomosis formation due to TRAP disorder. Malone FD et al. defined the twin-to-twin transfusion syndrome as an abnormality of the placental vascular architecture with arteriovenous communications that are not sufficiently balanced by arterioarterial and venovenous anastomoses [8]. Understanding this abnormal vascular formation that occurs in TRAP allows obstetricians and perinatologists to manage this condition with a less invasive method, such as radiofrequency thermal ablation of the umbilical cord of the acardiac fetus, rather than hysterotomy and removal of the acardiac twin [9, 10].

SUA occurs in approximately 0.5–5% of pregnancies screened antenatally [11]. SUA is considered a nonspecific marker, and when isolated, detection of SUA is associated with increased perinatal morbidity, such as fetal growth restriction, polyhydramnios, and oligohydramnios [12]. In addition, SUA may be associated with fetal chromosomal defects [13]. In TRAP, when there is reverse perfusion, the parasitic-twin steals poor oxygen blood from a provider twin through the umbilical artery (or arteries) and moves away through the umbilical vein, which is in the opposite direction to what usually occurs. There are no data on comparative analysis of TRAP cases with and without SUA. We hypothesized lower complication rates—such as FHF and preterm delivery—in pump twins when SUA is present. In our TRAP case, the presence of only one umbilical artery may limit the volume of blood reaching the acardiac twin, thus reducing the hemodynamic load on the pump twin's heart. Previously, the hemodynamic features of blood circulation in TRAP pregnancies have been discussed by Dashe et al., who revealed that the pump twins with poor pregnancy outcomes had a small resistive index between the pump and acardiac twin. Authors also found out in their TRAP series that poor outcomes were associated with larger acardiac twins (≥ 1100 g), smaller pump twins (≤ 2300 g), and higher twin weight ratios (≥ 48%) [14]. Additionally, one study has shown that a larger acardiac twin (often associated with normal two-artery cords) increases the hemodynamic burden on the pump twin [8]. Undesirable circulatory disorders that can develop in pump twins (cardiac failure, polyhydramnios, and hydrops, up to fetal loss) are not observed in the perinatal period in our case. Even in the 3-month-old and 6-month-old postpartum period, the child shows no cardiovascular pathologies. Normally, as we know, the umbilical cord consists of 2 umbilical arteries and 1 vein. In our case, postnatal macroscopic placental evaluation shows 2-vessel umbilical cord detection, later confirmed by the placenta's postpartum pathological examination. Notably, detecting an absent umbilical artery may indicate an additional screening for fetal anomaly due to association with concomitant fetal abnormalities. However, in the present report, an observation generates a hypothesis that the nonexistence of an impacted intrauterine fetal development and FHF of the provider twin may be explained by the presence of one umbilical artery, resulting in less blood flow to the parasitic twin, which can be accepted as a protective factor-effect for pump twin circulation in TRAP pregnancies.

MCMA pregnancies, especially if complicated by TRAP, require particular multidisciplinary approaches by perinatology and pediatric cardiology. Malone emphasized a vital need for large prospective studies for monochorionic gestation with late pediatric outcomes [8]. Prospective designed studies may potentially be helpful in the identification of incidence, natural history, long-term outcomes, and complications of monochorionicity. Our observation association between SUA and reduced hemodynamic load on the pump twin, which came across in our case report, is based on limited data and should be considered a preliminary finding.

## Conclusion

In conclusion, a Latin phrase comes to mind: " Procul dubio" meaning " Without a doubt." However, science develops with doubts, hypotheses, and sometimes errors; this is how reliable results and conclusions emerge. Our findings, which suggest that SUA may play a protective role in the development of FHF in pump twins, are promising but remain speculative, and further studies on this topic are needed to confirm the protective role of SUA for pump-twin circulation in TRAP pregnancies. In this case report, we would like to emphasize the need for additional research to validate our hypothesis, and we believe that multidisciplinary clinical collaboration will be useful in understanding TRAP-related complication.

## Data Availability

No datasets were generated or analysed during the current study.
